# An edge β‐strand mutation turns ubiquitin into a pore‐forming amyloid

**DOI:** 10.1002/pro.70603

**Published:** 2026-05-17

**Authors:** Daniela Meleleo, Rosanna Mallamaci, Vincenza Calò, Maurizio Losacco, Vincenzo Mangini, Fabio Arnesano

**Affiliations:** ^1^ Department of Science of Agriculture Food, Natural Resources and Engineering, University of Foggia Foggia Italy; ^2^ Department of Biosciences Biotechnologies and Environment, University of Bari “Aldo Moro” Bari Italy; ^3^ Department of Chemistry University of Bari “Aldo Moro” Bari Italy; ^4^ Institute of Crystallography, National Research Council Bari Italy

**Keywords:** β‐strand mutation, adhesin‐like motif, amyloid oligomers, antimicrobial peptides, ion channels, membrane permeabilization, ubiquitin

## Abstract

Ubiquitin (Ub) is a highly conserved eukaryotic protein, generally regarded as stable and soluble under physiological conditions, playing a key role in maintaining cellular protein balance. Using complementary bioinformatic, biophysical, immunochemical, and electrophysiological approaches, we show that a single point mutation—the substitution of Glu16 with Val (E16V) in an edge β‐strand—dramatically alters Ub behavior, inducing amyloid‐like aggregation, membrane permeabilization, and cytotoxicity. Remarkably, E16V retains the native globular fold of wild‐type Ub in aqueous solution, yet undergoes a functional switch upon interaction with anionic membranes. E16V assembles into prefibrillar oligomers, forms voltage‐dependent ion channels with well‐defined conductance states and lifetimes, and disrupts membrane integrity in both bacterial and mammalian cells. In contrast, wild‐type Ub remains monomeric and inert under identical conditions. Synthetic peptides encompassing the mutated β‐strand reproduce the cytotoxic effects, supporting a localized, sequence‐specific mechanism of action reminiscent of amyloidogenic motifs found in yeast adhesins. These findings uncover a hidden amyloidogenic potential in Ub and establish the E16V mutant as a unique model for membrane‐triggered amyloid pore formation and the rational design of membrane‐active antimicrobial peptides.

## 1 INTRODUCTION

Ubiquitin (Ub) is a small, highly conserved regulatory protein (Hershko & Ciechanover, 1998; Hochstrasser, 1996; Schlesinger & Goldstein, 1975) that plays pivotal roles in numerous cellular pathways (Pickart & Eddins, 2004; Pickart & Fushman, 2004). Its best‐characterized function is ubiquitination, a post‐translational modification in which one or more Ub molecules are covalently attached to substrate proteins, targeting them for degradation by the proteasome (Chau et al., 1989; Lemma et al., 2023; Tundo et al., 2020) or the autophagy‐lysosome system (Lauwers et al., 2009; Mizushima, 2024; Tan et al., 2008). These degradation pathways are essential for maintaining cellular proteostasis by removing misfolded or damaged proteins tagged with polyUb chains (Chiti & Dobson, 2017; Hipp et al., 2019; Le Guerroué & Youle, 2021; Liu & Nussinov, 2013; Munari et al., 2022).

A growing body of evidence implicates Ub in the pathogenesis of neurodegenerative disorders—including Parkinson's and Alzheimer's diseases—where Ub‐positive inclusions accumulate in neuronal tissues (Ciechanover & Brundin, 2003; Ross & Pickart, 2004). Impairment of the Ub–proteasome system (UPS) promotes the formation of toxic protein aggregates and contributes to cell death (Bellia et al., 2019; Kaganovich et al., 2008). Moreover, polyUb chains themselves can form amyloid‐like fibrils that act as initiation signals for autophagy (Morimoto et al., 2015).

Small soluble amyloid oligomers and prefibrillar intermediates are typically more cytotoxic than mature fibrils (Lashuel et al., 2002). Unlike stable fibrillar deposits, amyloid oligomers can permeabilize membranes either by forming ion‐conducting pores or through detergent‐like disruption (Demuro et al., 2005; Kayed et al., 2004; Nguyen et al., 2021; Zhao et al., 2012). Membrane lipid composition also modulates the interactions of peptides and small proteins with bilayers, as shown for Aβ peptides and human amylin (Meleleo et al., 2013; Meleleo & Picciarelli, 2016; Micelli et al., 2004; Sciacca et al., 2012; Zhang et al., 2017). Binding is typically initiated by electrostatic attraction (Byström et al., 2008), and negatively charged lipids strongly enhance the formation of toxic aggregates (Aisenbrey et al., 2008). In amyloidogenic proteins, membrane association increases local concentration and facilitates hydrophobic contacts, thereby accelerating self‐assembly (Aisenbrey et al., 2008; Seelig, 2004; Stefani, 2008).

Beyond its canonical roles in protein degradation, Ub and Ub‐derived peptides—either secreted into the extracellular milieu (Majetschak, 2011) or generated within the lysosomal compartment (Purdy & Russell, 2007b)—display antimicrobial activity against bacteria, fungi, and yeast (Alonso et al., 2007; De Ingeniis et al., 2009; Kieffer et al., 2003; Kim et al., 2007; Purdy & Russell, 2007a; Scavello et al., 2022). Although their mechanisms remain unclear, ultrastructural damage observed by electron microscopy resembles that induced by pore‐forming antimicrobial peptides such as defensins (Brogden, 2005; Foss et al., 2012; Kagan et al., 1990). The presence of a hydrophobic patch further suggests that Ub may directly interact with membrane lipids and possibly undergo passive internalization (Mendoza‐Salazar et al., 2024).

Given that membrane disruption is a shared hallmark of both amyloid toxicity and antimicrobial action (Arispe et al., 1993; Butterfield & Lashuel, 2010; Kagan et al., 2012; Yoshiike et al., 2007), we examine the aggregation propensity of wild‐type (WT) Ub and selected point mutants at the membrane interface. In particular, we identify a single substitution within an edge β‐strand that preserves the native fold in solution but triggers aggregation upon exposure to anionic membranes. To elucidate the structural and functional impact of this mutation, we integrate biophysical, electrophysiological, and cellular assays to reveal how localized surface changes modulate Ub–membrane interactions and potentially drive cytotoxic or antimicrobial outcomes.

## 2 RESULTS

### 2.1 Aggregation propensity of Ub mutants

We analyzed the aggregation propensity of WT Ub and all possible single‐point mutants using TANGO, an algorithm developed by Serrano and co‐workers (Fernandez‐Escamilla et al., 2004), which predicts β‐aggregation‐prone regions and their enhancement upon specific amino acid substitutions. As expected, no aggregation tendency was predicted for WT Ub. In contrast, TANGO identified a markedly increased aggregation propensity when Glu16—and, to some extent, Lys6, Lys11, His68, or Arg72—was replaced by a hydrophobic residue (Figure S1a). In particular, substitution of Glu16 with valine (E16V), located in the edge β‐strand, strongly enhanced β‐aggregation propensity. Notably, replacement of the nearby Glu18 (in a loop region) with valine (E18V) had no such effect, underscoring the critical role of the edge β‐strand in promoting aggregation susceptibility.

Based on TANGO analysis predictions, we expressed WT Ub and the E16V mutant. The cDNA sequences encoding these proteins were cloned into pET‐3a expression vectors and used to transform *Escherichia coli* BL21(DE3) cells. Expression of the E16V mutant resulted in a marked decrease in *E. coli* cell viability (Figure S1b).

To investigate the intracellular distribution of the proteins, soluble and insoluble fractions of *E. coli* lysates were analyzed by Western blot using anti‐Ub antibodies. WT Ub was detected mainly in the soluble fraction, whereas E16V was distributed across all fractions, including membrane‐associated ones. While a small insoluble fraction can occasionally be detected for WT Ub, aggregation is markedly enhanced for the E16V variant under identical conditions. Notably, a high molecular weight smear consistent with oligomeric species was observed in the membrane fraction of E16V‐expressing cells (Figure S1c).

**FIGURE 1:**
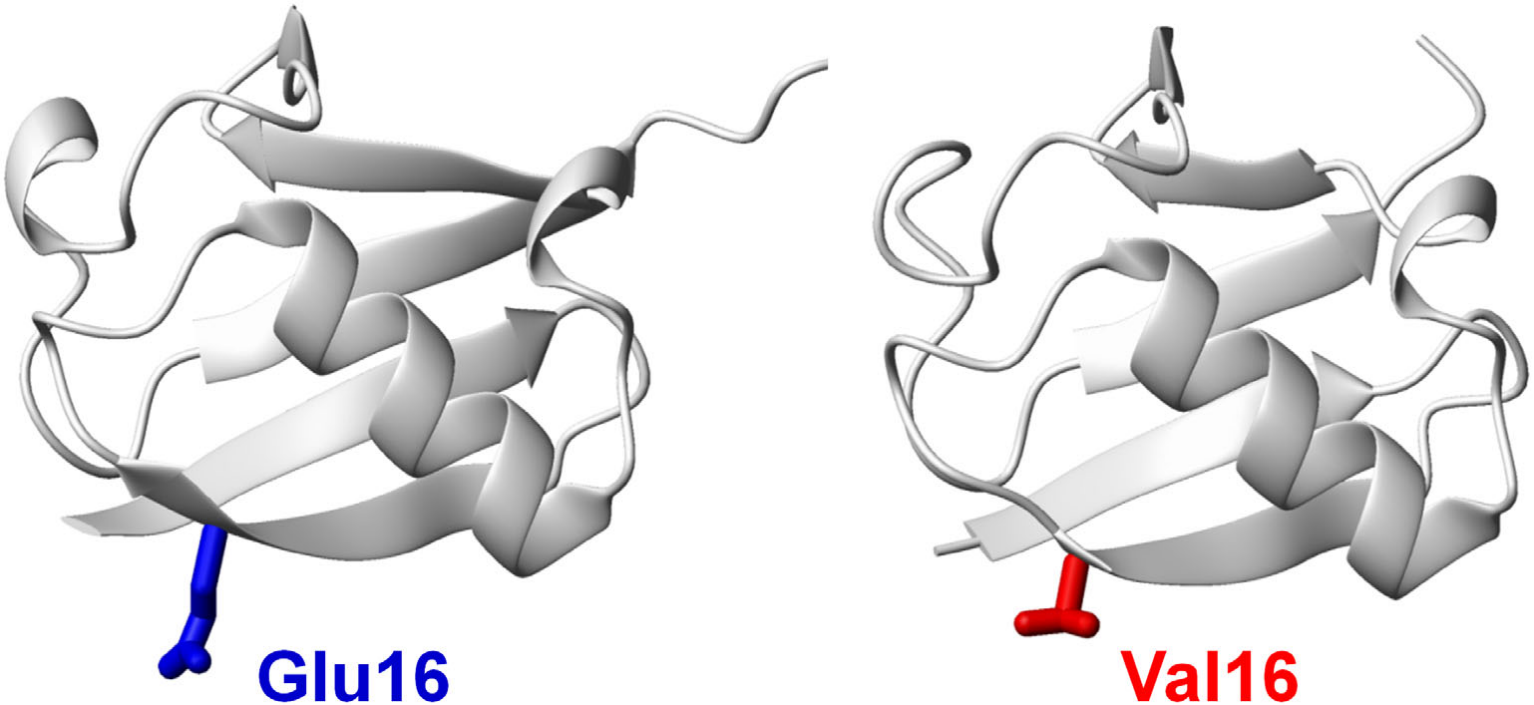
X‐ray crystal structures of human ubiquitin. Structures of wild‐type ubiquitin (*left*; PDB ID 1UBQ) (Vijay‐Kumar et al., 1985) and the E16V mutant (*right*; PDB ID 5NL4) (Fermani et al., 2018) are shown for comparison.

Our previous study demonstrated that, in aqueous solution, WT Ub and the E16V mutant display remarkably similar structures, including at the mutation site, as confirmed by NMR spectroscopy and X‐ray crystallography (Fermani et al., 2018) (Figure 1). Despite their structural similarity and comparable stability in solution, interactions with lipid membranes can induce conformational changes and promote aggregation, depending on the membrane's lipid composition.

### 2.2 Conformational changes of E16V on anionic phospholipid bilayers

The above observations prompted us to examine the effects of the purified proteins on model membranes, using phospholipid liposomes as biomimetic systems.

To this aim, liposomes composed of 800 μM phosphatidyl‐serine (PS, anionic) or phosphatidylcholine (PC, zwitterionic) were prepared and added to a solution containing 10 μM WT or mutant Ub. The mixtures were incubated at 37°C in phosphate buffer (pH 7.4), and changes in protein secondary structure induced by the lipid bilayers were monitored over time using circular dichroism (CD) spectroscopy.

**FIGURE 2:**
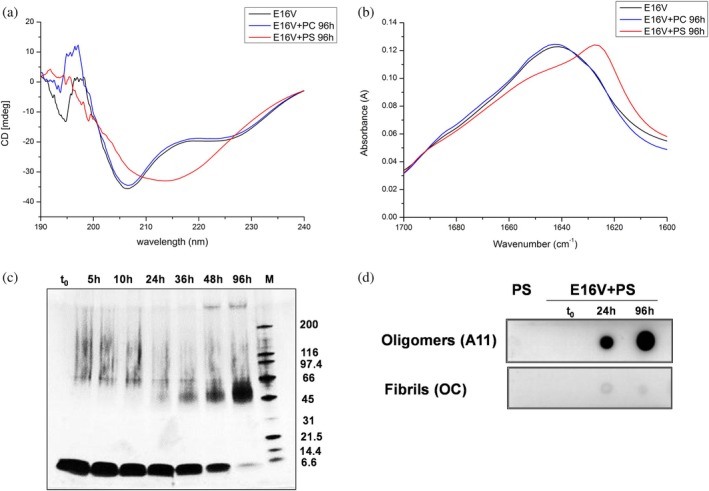
Structural and aggregation properties of E16V in the presence of lipid membranes. (a) Far‐UV circular dichroism (CD) spectra of E16V (10 μM) alone (black) or in the presence of 800 μM phosphatidylserine (PS, red) or phosphatidylcholine (PC, blue) liposomes in phosphate buffer (pH 7.4) at 37°C. (b) ATR‐FTIR spectra in the amide I region of E16V under the same conditions. (c) SDS‐PAGE analysis of E16V (80 μM) incubated with PS liposomes (800 μM) at 37°C at increasing incubation times. (d) Dot blot analysis of E16V incubated with PS liposomes, probed with conformation‐specific A11 (anti‐oligomer) and OC (anti‐fibril) antibodies. PS liposomes without protein were used as control.

In the presence of anionic liposomes, no significant changes were observed in the CD profile of WT Ub (Figure S2a), whereas E16V displayed a marked alteration consistent with increased β‐sheet content (Figure 2a).

Notably, the control mutant E18V, in which the same substitution is shifted two residues downstream to a loop region, did not undergo time‐dependent conformational changes in the presence of PS liposomes (Figure S2c). Because E16V and E18V carry the same Glu → Val substitution and therefore the same theoretical pI shift relative to WT Ub, this observation indicates that the liposome‐induced structural conversion in E16V depends on the specific position of the mutation within the edge β‐strand.

This structural transition was further confirmed by Attenuated Total Reflectance Fourier Transform Infrared (ATR‐FTIR) spectroscopy through analysis of the amide I region (Figure S2b and Figure 2b), where the appearance of a band at 1625 cm^−1^ in the E16V + PS sample indicates the formation of β‐sheet‐rich structures typical of amyloid aggregates. In contrast, zwitterionic PC liposomes did not induce comparable structural changes in any of the tested proteins, including E16V (Figure 2a,b).

The aggregation propensity of 10 μM E16V was further assessed by sodium dodecyl sulfate polyacrylamide gel electrophoresis (SDS‐PAGE). While WT Ub remained predominantly monomeric, incubation of E16V with PS liposomes resulted in the disappearance of the monomeric band and the appearance of a broad distribution of higher molecular weight species (Figure S3). Consistently, time‐dependent analysis at higher protein concentration revealed the progressive formation of oligomeric species, with the appearance of a band between 45 and 66 kDa (Figure 2c).

These aggregated species were characterized by immunochemical assays with conformation‐dependent antibodies that recognize structural epitopes specific to distinct amyloid aggregation states (Glabe, 2008). The A11 antibody selectively detects prefibrillar amyloid oligomers, but not monomers, fibrils, or natively folded proteins (Kayed et al., 2003). In contrast, the OC antibody specifically binds fibrils but not oligomers (Kayed et al., 2007), indicating that these species represent conformationally distinct amyloid forms.

A dot blot assay was performed at different time points on samples of PS alone and E16V + PS. The aggregates formed in the E16V + PS mixture were recognized by A11, but not by OC, indicating that they are prefibrillar oligomers (Figure 2d).

### 2.3 Membrane binding and permeabilization by E16V amyloid oligomers

**FIGURE 3:**
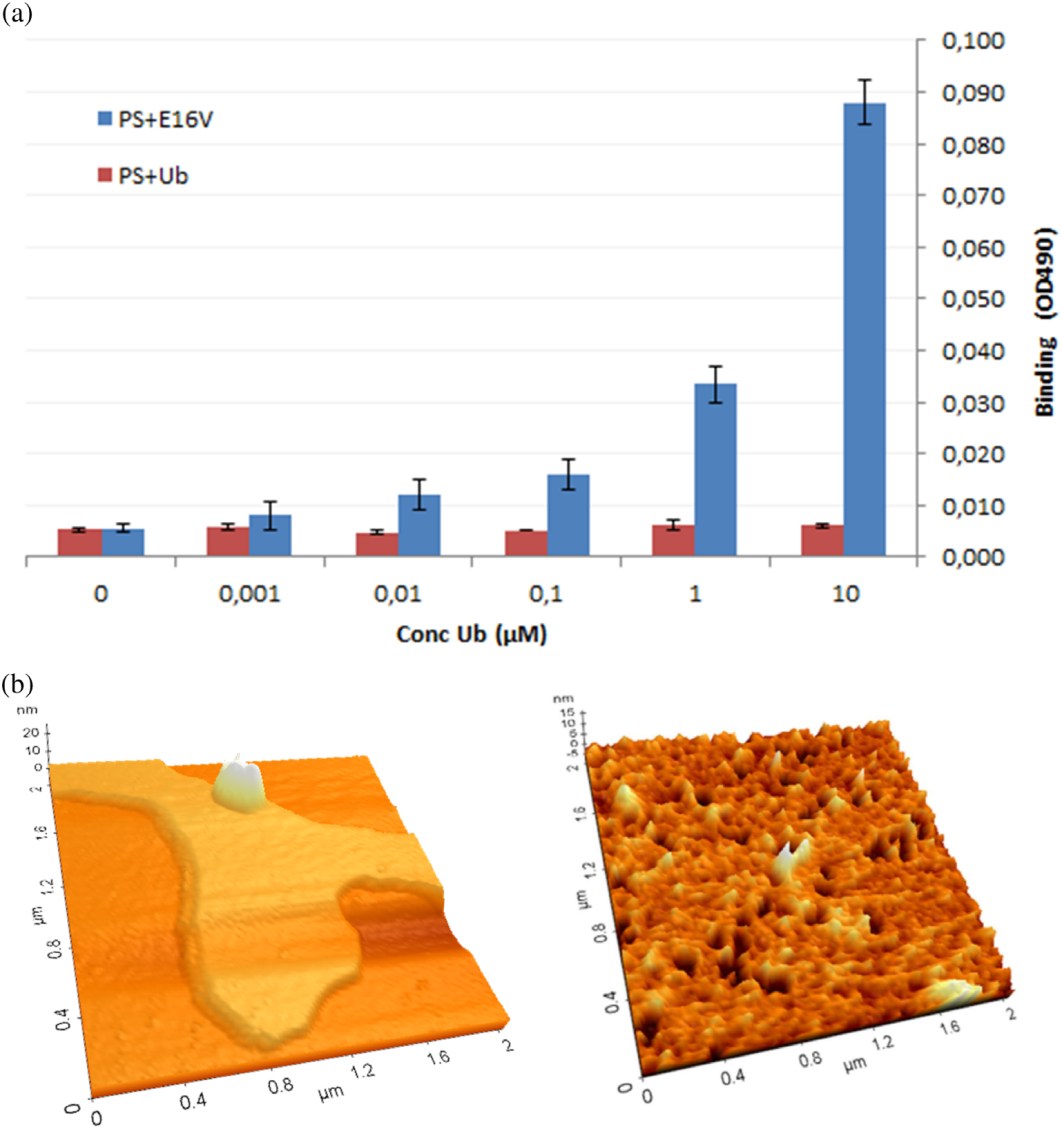
Interaction of ubiquitin and its variant with anionic membranes. (a) Binding of wild‐type ubiquitin (WT Ub, red) and E16V (blue) to PS lipids immobilized on microplates. Experiments were performed in PBS. Data are shown as mean ± SD (*n* = 2). (b) Topographic AFM images of PS planar bilayers in the absence (*left*) and presence (*right*) of E16V.

WT Ub and E16V, incubated with lipid membranes, were subjected to a solid‐phase binding assay to assess their interaction with anionic phospholipid liposomes immobilized on microplates, using anti‐Ub antibodies for detection. The assay revealed that only E16V binds strongly to anionic liposomes (Figure 3a). Notably, the solid‐phase binding assay was performed in PBS, that is, under physiological ionic strength conditions, indicating that E16V retains strong interaction with anionic liposomes also at physiological salt concentrations. This result suggests that the β‐structural transition of E16V is linked to its strong affinity for anionic liposomes, possibly reflecting the formation of membrane‐associated intermediates.

To assess the stability of these E16V oligomeric species formed in the presence of anionic bilayers, we tested their solubility using the zwitterionic detergent lauryldimethylamine oxide (LDAO). Addition of LDAO to E16V oligomeric samples, previously converted into β‐structured species, led to a rapid return to the monomeric state, as shown by CD and ATR‐FTIR analyses (Figure S4). This result indicates that the E16V oligomers lack the strong, irreversible cross‐β‐sheet interactions typical of highly stable amyloid fibrils. The disruption by LDAO suggests that oligomer stabilization involves surface charge and hydrophobic interactions. The reversibility of the process supports the hypothesis that E16V promotes dynamic, membrane‐associated oligomers, which may underlie its membrane‐permeabilizing and potentially cytotoxic behavior.

The ability of WT Ub and E16V to permeabilize anionic membranes was evaluated using a relaxometric approach based on the measurement of the longitudinal relaxation time (*T*_1_) of bulk water protons in liposome suspensions encapsulating a high concentration of Gd(III) complex in the aqueous lumen. The paramagnetic relaxation enhancement observed in the bulk water reflects water permeability across the liposomal bilayer and is quantified using the specific relaxivity per unit Gd (*R*_1p_).

Liposomes composed of dipalmitoylphosphatidylcholine (DPPC) and dipalmitoylphosphatidylserine (DPPS) at a 50:50 molar ratio were incubated with WT Ub and E16V. The results show a marked increase in *R*_1p_ values for liposomes treated with E16V compared to those incubated with WT Ub, indicating that E16V enhances water permeability of the liposomal membrane (Figure S5).

To further assess the impact of E16V on membrane integrity, atomic force microscopy (AFM) was used to visualize a planar bilayer formed from PS liposomes, either alone or in the presence of E16V. PS liposomes were deposited onto freshly cleaved mica and allowed to fuse and rupture, forming a homogenous lipid bilayer with a thickness of 4.5–5.0 nm (Figure 3b, *left*). When the same procedure was applied to samples containing both E16V and PS liposomes, AFM images revealed a sponge‐like morphology of the bilayer characterized by the presence of discrete pores. These features suggest that E16V disrupts membrane integrity by interacting with and inserting into the lipid bilayer (Figure 3b, *right*).

Taken together, these experiments indicate that E16V behaves like an amyloidogenic protein, capable of forming β‐sheet‐rich, lipid‐interacting oligomers that destabilize the membrane structure.

### 2.4 Channel activity of E16V in planar lipid membranes

The behavior of WT Ub and E16V toward planar lipid membranes (PLMs) composed of palmitoyl‐oleoyl‐phosphatidylserine (POPS), an anionic phospholipid typical of cellular membranes, was evaluated using single‐channel current recordings.

**FIGURE 4:**
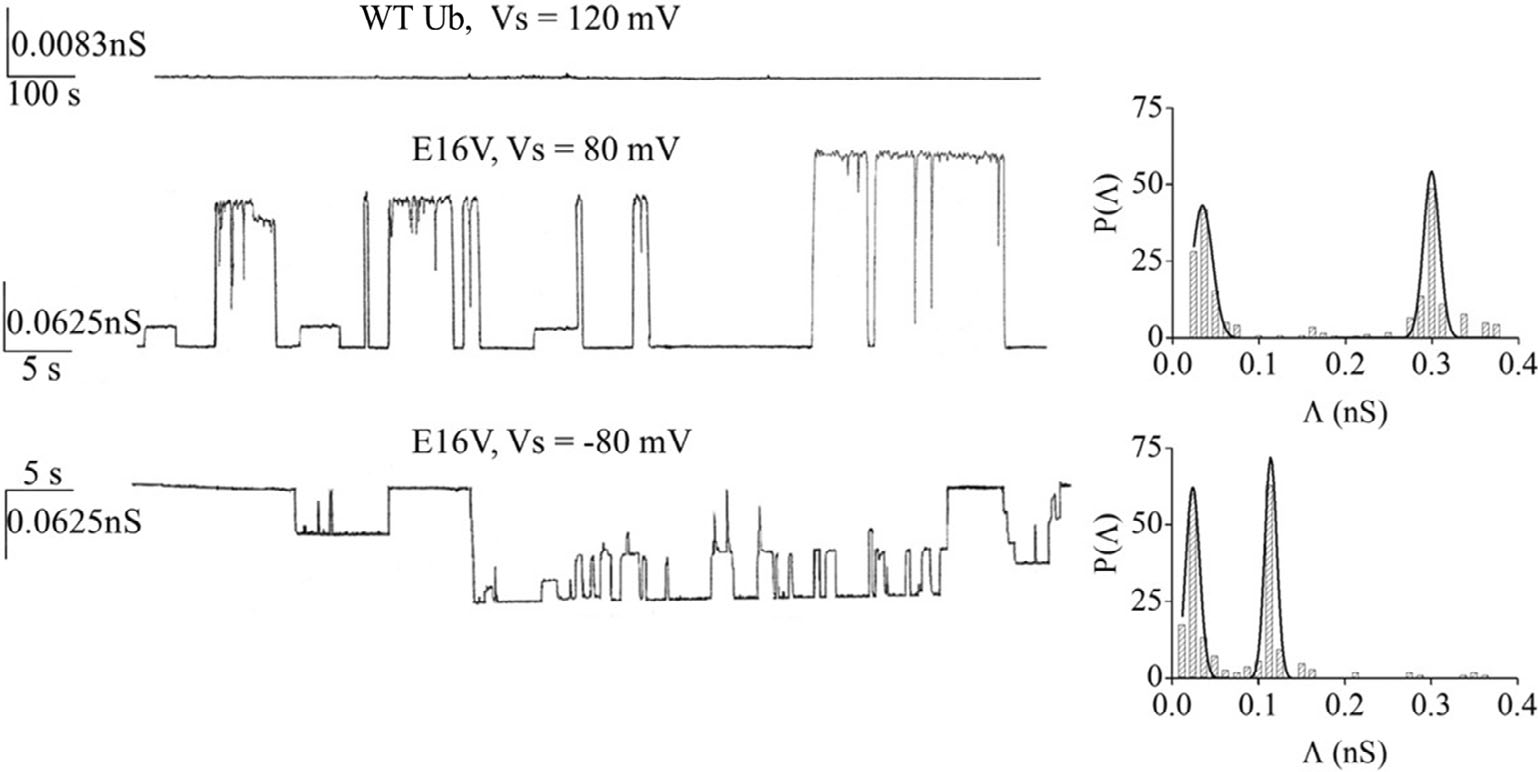
Channel activity of E16V in planar lipid membranes (PLMs). Representative single‐channel current recordings of wild‐type ubiquitin (WT Ub) and E16V (1 μM) in PLMs composed of POPS, recorded at the indicated applied voltages (Vs). *Left*: Representative current traces showing the absence of channel activity for WT Ub and the appearance of discrete channel events following E16V addition. *Right*: Corresponding conductance histograms of individual E16V channel events, expressed as the probability P(Λ) of observing a given conductance value. Solid curves represent Gaussian fits used to resolve distinct conductance populations. Recordings were performed in 1 M KCl (pH 7.0) at 23°C ± 1°C.

Addition of WT Ub produced no detectable changes in membrane conductance, even at applied voltages up to ±120 mV (Figure 4). After 20 min, however, a modest increase in membrane capacitance was observed (from 0.35 to 0.42 μF/cm^2^), consistent with protein adsorption and thinning of the bilayer, but no conductive events were detected.

In contrast, addition of E16V led to clear channel activity following a lag phase of approximately 1 h. Recordings displayed sudden and stochastic current jumps between discrete open and closed states, often occurring in clusters of bursts separated by quiescent periods, characteristic of dynamic pore formation by oligomeric species (Figure S6).

Amplitude histograms derived from single‐channel current recordings revealed two predominant conductance levels, Level 1 and Level 2, which were resolved by Gaussian fitting (Figure 4). The resulting central conductance values, expressed as mean conductance ± standard error (Λ_c_ ± SE), for each channel population at different voltages are summarized in Table S1. Λ_c_ values were consistently higher at positive than at negative voltages for both populations, indicating a charge asymmetry across the two sides of the conductive unit.

A kinetic analysis, including open probabilities and lifetime distributions for both conductance levels, is provided in Supplementary Methods Table S1. These data indicate that E16V channels display longer‐lived conductive states at positive voltages, consistent with a greater kinetic stability under positive voltage conditions.

Using Λ_c_ values and the standard cylindrical pore approximationc (Supplementary Methods Table S1), the pore diameters for the two channel populations were estimated to be 1.4/1.2 Å (Level 1) and 4.1/2.6 Å (Level 2) at +80/−80 mV, respectively.

Together, these results demonstrate that E16V, but not WT Ub, inserts into POPS bilayers and assembles into discrete conductive units.

### 2.5 Characterization of the E16V ion channel

The current–voltage (*I*–*V*) relationship of the E16V channel in the open state is ohmic, displaying a linear current–voltage dependence (Figure S7) with a single‐channel conductance of 0.36 nS.

Biophysical and statistical parameters of E16V channel activity at different protein concentrations are summarized in Table S2. At low concentrations (0.01 and 0.1 μM), channel activity consisted of multi‐level current fluctuations with brief openings and closures (*multistep‐channel activity*), consistent with progressive assembly of conductive units within the membrane following adsorption and insertion. At higher concentrations (1 and 3 μM), activity was dominated by abrupt, well‐defined stepwise transitions between discrete open and closed states (*step‐channel activity*; see Figure S8), consistent with insertion of preassembled oligomeric units and formation of more stable conductive assemblies.

As reported for other proteins (Bertrand et al., 2021), heterogeneous current traces may reflect distinct pore conformations or oligomeric states; nonetheless, the observation of discrete, well‐defined conductive units provides strong evidence for E16V aggregation.

### 2.6 Cellular toxicity of E16V and its amyloidogenic region

The cytotoxicity of WT Ub and E16V was evaluated by incubating each protein with HeLa cells. E18V, which carries the same mutation at a slightly different position in the sequence, was also expressed and tested. Cell‐based assays were performed in DMEM culture medium, which contains physiological salt concentrations, providing conditions close to the physiological environment. Cell death was assessed using the fluorescent probe ethidium homodimer‐1, which penetrates damaged cell membranes and binds to nucleic acids, producing bright red fluorescence in non‐viable cells.

**FIGURE 5:**
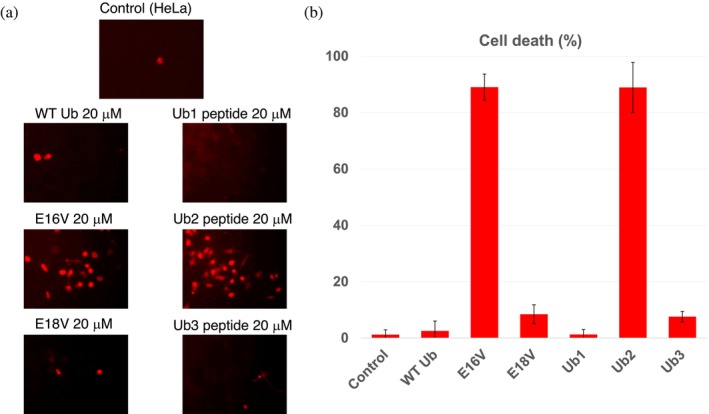
Cytotoxic effects of ubiquitin variants and derived peptides. (a) Representative fluorescence images of HeLa cells treated with WT Ub, E16V, or E18V (*left*), and with synthetic peptides Ub1, Ub2, or Ub3 (*right*). Dead cells are visualized by ethidium homodimer‐1 staining (red). Experiments were performed in DMEM under physiological conditions. (b) Quantification of cell death expressed as the percentage of ethidium‐positive cells relative to the total cell population. Data are presented as mean ± SD from two independent fields of view analyzed with ImageJ (Schindelin et al., 2012).

A significant increase in cell mortality was observed only in samples treated with E16V, whereas cells exposed to WT Ub or E18V remained largely viable (Figure 5).

Previous studies have reported that N‐ and C‐terminal peptides of Ub possess antimicrobial activity by interacting with cell membranes (Kieffer et al., 2003; Kim et al., 2007), and that cell adhesion molecules often contain functional amyloid‐forming sequences (Siemer, 2022). In particular, Ramsook et al. identified β‐aggregation‐prone motifs in yeast adhesins, characterized by an unusual enrichment in branched aliphatic amino acids, such as isoleucine, threonine, and valine (Ramsook et al., 2010).

In light of these findings, the amino acid sequence of human Ub was analyzed for the presence of similar motifs. A heptapeptide region spanning residues 12–18 (TITLEVE) was identified, containing multiple branched aliphatic residues and closely resembling the β‐aggregation‐prone sequences described in yeast adhesins (Ramsook et al., 2010). Both E16V and E18V mutations increase the content of branched aliphatic amino acids within this region; however, only E16V generates a continuous stretch of such residues, whereas E18V retains Glu16, which disrupts the adhesin‐like motif.

To evaluate the potential cytotoxicity of these sequence variants, three synthetic peptides were prepared: Ub1 (TITLEVE), Ub2 (TITLVVE), and Ub3 (TITLEVV), corresponding to the WT, E16V, and E18V sequences, respectively. Cell viability assays in HeLa cells showed that only Ub2, derived from the E16V variant, induced a marked increase in cell death, whereas Ub1 and Ub3 exhibited only minimal toxicity (Figure 5).

These results indicate that E16V exhibits structural and functional features reminiscent of adhesins, as it forms β‐aggregation‐prone, membrane‐interacting oligomers enriched in branched aliphatic residues, consistent with the amyloidogenic motifs described in cell surface proteins. The absence of such behavior in E18V, which lacks a continuous stretch of these residues, indicates that the specific point of mutation is critical for membrane disruption and cytotoxicity.

## 3 DISCUSSION

Ubiquitin is widely used as a model system for stability, folding, and structural studies (Jackson, 2006; Job et al., 2015). It plays a central role in both the proteasomal and lysosomal pathways, where mono‐ and polyubiquitination function not only as degradation signals but also regulate intracellular trafficking (Bunker et al., 1987) and modulate innate immune responses (Hicke, 2001; Jiang & Chen, 2011; Metz‐Boutigue et al., 2003; Nabika et al., 1999). Owing to its essential physiological roles, Ub is highly conserved and typically exhibits remarkable structural stability, although specific environmental conditions can promote its destabilization.

Previous NMR studies demonstrated that heavy‐metal ions such as Cu^2+^ and Zn^2+^ bind to an aggregation‐prone region at the N‐terminus of Ub, with Glu16 acting as a coordinating ligand (Arena et al., 2011; Arnesano et al., 2011; Fermani et al., 2013), and such metal binding promotes oligomerization (Arena et al., 2013; Arnesano et al., 2009). Guided by the statistical‐mechanics algorithm TANGO, we designed mutations to mimic the charge‐neutralizing effect of metal‐carboxylate coordination. Two Ub variants were generated in which Glu was replaced by Val: E16V within an edge β‐strand and E18V within a loop region.

CD and NMR analyses confirmed that both mutants remain soluble and well folded in solution, similar to WT Ub (Fermani et al., 2018). However, only E16V undergoes time‐dependent conformational rearrangements upon incubation with anionic phospholipid liposomes, accompanied by an increase in β‐sheet content detected by CD and ATR‐FTIR spectroscopy. Lipid‐soluble β‐rich oligomers form, penetrate the bilayer, and react with the A11 antibody, which selectively recognizes toxic amyloid oligomers. These species are dynamic assemblies stabilized by weak non‐covalent interactions and dissociate in the presence of a zwitterionic detergent, which restores the monomeric state of E16V. Notably, amyloidogenic behavior is observed only in the presence of anionic, not zwitterionic, liposomes.

Both single‐channel current recordings and relaxometric assays indicate that E16V increases the permeability of anionic phospholipid bilayers. E16V also forms oligomeric species in the insoluble fractions of *E. coli* and shows cytotoxicity toward eukaryotic cells. The underlying mechanism involves a β‐aggregation‐prone motif (residues 12–18), enriched in branched aliphatic residues (Ile, Val, Thr), reminiscent of amyloidogenic sequences in yeast adhesins (Ramsook et al., 2010). Consistently, only the E16V‐derived heptapeptide (Ub2: TITLVVE) was cytotoxic to HeLa cells, whereas WT Ub‐ and E18V‐derived peptides showed minimal toxicity.

The ability of E16V to form amyloid‐like, pore‐forming oligomers suggests that this variant provides a valuable model for investigating mechanisms relevant to neurodegeneration. Furthermore, amyloid formation by Ub may contribute to its antimicrobial activity, a process facilitated by metal ions and by interactions with the anionic phospholipid bilayers characteristic of prokaryotic membranes.

Several Ub‐derived peptides exhibit antimicrobial activity through distinct mechanisms: N‐ and C‐terminal fragments act synergistically to inhibit fungal growth via calcineurin targeting (Alonso et al., 2007; Kieffer et al., 2003; Kim et al., 2007), whereas peptides produced by lysosomal processing show membrane‐permeabilizing effects, particularly against *Mycobacterium tuberculosis* (Foss et al., 2012; Gutsmann, 2016). A secreted Ub‐like protein from *Pichia anomala* also displays broad‐spectrum bactericidal activity (De Ingeniis et al., 2009), suggesting that full‐length Ub harbors an intrinsic antimicrobial potential.

Within this framework, our study shows that a single β‐strand mutation in full‐length Ub is sufficient to activate this potential, promoting amyloid‐like aggregation, membrane permeabilization, and ion‐channel formation in a native structural context. This behavior parallels the structural plasticity of chloride intracellular channel (CLIC) proteins, which can transition from soluble cytosolic forms to membrane‐integrated ion channels (Littler et al., 2010) and establishes that a globular eukaryotic protein can acquire pore‐forming activity through a minimal surface perturbation, without proteolytic processing.

Taken together, our findings support a model in which local charge neutralization—by mutation or metal binding—combined with interactions with anionic lipids can unmask an intrinsic amyloidogenic and pore‐forming capacity in Ub, providing new insight into its potential roles in cytotoxic, antimicrobial, or stress‐related membrane‐disruptive processes.

## 4 MATERIALS AND METHODS

### 4.1 TANGO analysis

All possible single‐point mutants of Ub were generated in silico by systematically replacing each residue in the sequence with the other 19 standard amino acids, yielding a total of 1444 variants. These sequences were analyzed using the TANGO algorithm, which predicts aggregation‐prone regions based on the statistical mechanics of β‐structure formation (Fernandez‐Escamilla et al., 2004). Calculations were performed at pH 7, 298 K, and an ionic strength of 0.02 M. TANGO assumes the polypeptide is unfolded and solvent‐exposed. While these conditions do not reflect the native folded state of Ub, they allow prediction of aggregation‐prone regions that may become exposed upon environmental perturbation or membrane interaction.

### 4.2 Chemicals

Potassium chloride (KCl) and *n*‐decane were purchased from Sigma (Munich, Germany). l‐α‐Phosphatidyl‐l‐serine (PS) from *Glycine max* (soybean) and l‐α‐phosphatidylcholine (PC) were also purchased from Sigma. Palmitoyl‐oleoyl‐phosphatidylserine (POPS), palmitoyl‐oleoyl‐phosphatidylcholine (POPC), dipalmitoyl‐phosphatidylserine (DPPS), and dipalmitoyl‐phosphatidylcholine (DPPC) were purchased from Avanti Polar Lipid (Alabaster, AL). Synthetic peptides were obtained from GenScript (Piscataway, NJ).

All solutions were prepared using ultrapure Milli‐Q water from a Millipore purification system.

### 4.3 Protein expression and purification

The full‐length WT Ub, E16V, and E18V genes cloned into pET‐3a vectors were expressed in *E. coli* BL21(DE3) cells. Proteins were purified by selective precipitation with perchloric acid, followed by cation exchange chromatography (HiPrep SP FF 16/10) and size exclusion chromatography (Superdex 75 10/300 GL). Complete buffer salt removal was achieved using the HiTrap Desalting column. All chromatographic steps were performed on an ÄKTA Purifier system (Amersham Biosciences). Purified proteins were lyophilized using a Lio 5P freeze‐drier. Protein purity was confirmed by sodium dodecyl sulfate‐polyacrylamide gel electrophoresis (SDS‐PAGE) and electrospray ionization mass spectrometry (ESI‐MS).

Protein solutions were prepared by dissolving the lyophilized material in ultrapure Milli‐Q water (Millipore purification system).

### 4.4 Western blotting

The aggregation of E16V was evaluated by Western blotting. Protein samples were separated by electrophoresis on Any kD precast TGX gel (Bio‐Rad) and subsequently transferred onto polyvinylidene difluoride (PVDF) membrane (Bio‐Rad). Membranes were blocked for 1 h at room temperature with 10% non‐fat dry milk in TBS‐T (Tris‐buffered saline with 0.1% Tween‐20), washed 3 times for 10 min each, and incubated overnight at 4°C with the primary anti‐oligomer A11 antibody (Invitrogen, 1:2000 in 5% non‐fat dry milk/TBS‐T) (Kayed et al., 2003). After additional washing steps (3 × 10 min), membranes were incubated for 1 h at room temperature with a goat anti‐rabbit IgG‐HRP conjugated secondary antibody (Santa Cruz Biotechnology, 1:5000 in 5% non‐fat dry milk/TBS‐T) and washed 3 × 10 min. Signal detection was performed using ECL chemiluminescence reagents (Thermo Scientific), and membranes were exposed to Hyperfilm™ (Amersham Biosciences).

### 4.5 Preparation of liposomes

Phosphatidylserine (PS) or phosphatidylcholine (PC) was dissolved in chloroform (40 mg/mL) and dried under a flow of dry N_2_ to form a thin lipid film. The film was vacuum‐desiccated for 30 min, then resuspended in 10 mM phosphate buffer (pH 7.4). The resulting suspension was vortexed for 30 min and sonicated in an ice bath using a titanium tip, promoting the formation of liposomes, whose diameter was estimated to be approximately 100 nm using dynamic light scattering.

### 4.6 Far‐UV circular dichroism

CD spectra were acquired using 10 μM protein solutions with 800 μM PC or PS liposomes in 1 mM phosphate buffer (pH 7.4). Far‐UV CD measurements were performed in low‐ionic‐strength phosphate buffer to allow reliable detection in the far‐UV region. Aggregation was initiated by incubation at 37°C. Spectra were recorded with a Jasco J‐810 spectropolarimeter using a 0.1 cm pathlength quartz cuvette, across a wavelength range of 190–250 nm, at a 0.1 nm data pitch. Each spectrum represents an average of 8 scans, baseline corrected and smoothed using adjacent averaging or FFT filtering.

### 4.7 Attenuated Total reflectance Fourier transform infrared spectroscopy

ATR‐FTIR spectra were recorded using a Varian IR‐670 spectrometer equipped with a DTGS detector operating in ATR mode and purged with a continuous flow of nitrogen. Spectra were collected at 25°C with 64 scans per sample, at a resolution of 4 cm^−1^, over the range 7900–375 cm^−1^. Each sample contained 10 μM protein and 800 μM phospholipids in 1 mM phosphate buffer (pH 7.4). A 10 μL aliquot was applied to a one‐reflection diamond prism (MIRacle ATR accessory, Pike technologies, WI). Spectra were recorded after drying under nitrogen, and buffer spectra were subtracted accordingly.

4.8

AFM imaging was performed using a PSIA XE‐100 SPM system operating in tapping mode in air, with a silicon nitride tip (MLCT‐AUHW, Park Scientific) mounted on a cantilever with a spring constant of 0.01 N m^−1^. Aliquots (15 μL) of sample were deposited onto freshly cleaved mica (NanoAndMore GmbH), allowed to dry in air for 2 min at room temperature, rinsed with MilliQ water, and gently dried with a nitrogen stream. Images were acquired in both height and phase modes at scan rates of 0.5–1 Hz with a resolution of 512 × 512 pixels. Image processing and analysis were performed using XEI software with plane adjustment.

### 4.9 Gel electrophoresis

WT Ub and E16V aggregation were assessed by SDS‐PAGE using 4%–20% gradient precast TGX gels (Bio‐Rad). Protein samples were mixed with Laemmli buffer, boiled for 5 min at 95°C, and resolved at 150 V in TGS running buffer. Gels were visualized by silver nitrate staining.

### 4.10 Immunoblotting and dot blotting

Aliquots (2 μL) of 10 μM protein solutions were spotted onto 0.45 μm nitrocellulose membranes (Bio‐Rad) and air‐dried for 15 min. Membranes were blocked with 10% non‐fat dry milk in TBS‐T (20 mM Tris, 0.8% NaCl, 0.01% Tween‐20, pH 7.6) for 1 h and incubated overnight at 4°C with A11 anti‐oligomer primary antibody (Invitrogen, 1:2000 in 5% milk/TBS‐T) (Kayed et al., 2003). After three 10‐min washes, membranes were incubated with goat anti‐rabbit IgG‐HRP conjugate (Santa Cruz Biotechnology, 1:5000 in 5% milk/TBS‐T) for 1 h, washed, and developed with ECL reagents (Thermo Scientific). Detection was performed on Hyperfilm (Amersham Biosciences). The same procedure was used for dot blotting with OC anti‐fibril antibody (1:10,000) (Kayed et al., 2007), kindly provided by Prof. Charles G. Glabe (University of California).

### 4.11 Solid‐phase binding assay

Pre‐formed liposomes were dissolved in ethanol (50 μg/mL) and coated onto 96‐well microtiter plates by evaporation. Wells were blocked for 2 h with 10% BSA in PBS. WT Ub or E16V at varying concentrations was added, and the plates were incubated for 96 h at 25°C. After washing with PBS, bound protein was detected using a mouse anti‐Ub antibody (Sigma–Aldrich, 1:1000), followed by HRP‐conjugated anti‐mouse IgG (1:5000). All washing and incubation steps were performed in PBS to maintain physiological ionic strength. Colorimetric detection was performed using O‐phenylenediamine as substrate and measured at 490 nm with a microplate reader (Molecular Devices). Specificity was confirmed with appropriate negative and positive controls.

### 4.12 Single‐channel measurements

Channel activity was assessed using planar lipid bilayer membranes (PLMs) composed of POPS in 1% *n*‐decane. The experimental approach followed established protocols (Meleleo et al., 2022; Meleleo & Picciarelli, 2016). Briefly, PLMs were formed across a 300 μm hole in a Teflon partition separating two chambers, *cis* and *trans*. The *cis* side contained either WT Ub or E16V, and voltage polarity was defined relative to this side. A negative potential was applied to the *trans* compartment, opposite to the *cis* side.

PLMs were formed using the Müller–Rudin or painted technique (Müller et al., 1962; Tien, 1974; Tien et al., 1977). Ionic currents were recorded on a chart recorder as previously described (Meleleo, 2021) and subsequently analyzed. Membrane capacitance was estimated using a calibration curve (Meleleo et al., 2023). All current traces were low‐pass filtered at 300 Hz, in line with standard practice (Arispe & Doh, 2002). Data were obtained from a minimum of four independent experiments conducted on different days.

WT Ub or E16V were tested at a final concentration of 1 μM over a voltage range of ±40 to ±80 mV. In addition, E16V was examined at different concentrations (0.01, 0.1, and 3 μM) to assess dose‐dependent effects. In these experiments, channel activity was monitored for up to 2 h under an applied voltage of 80 mV.

The following parameters were evaluated:

- Lag time: the duration between protein addition and the onset of channel activity.
- Conductance (Λ_c_): obtained from the amplitude of channel openings. Distributions were plotted as histograms and fitted to Gaussian functions; results are reported as Λ_c_ ± SE.
- Open probability (*P*_o_): the fraction of time the channel spends in a conductive state, calculated as the ratio of the total open time to the total recording time (Colquhoun & Sigworth, 1995; Laver, 2001).
- Frequency (*F*): the average number of channel opening events per 60 s, expressed as mean ± SD.
- Current–voltage (*I*–*V*) relationship: determined by plotting single‐channel current amplitudes recorded at different applied voltages (Supplementary Methods Table S1).
- Channel lifetime: duration of individual open events, defined as the time interval between channel opening and closing transitions (see Appendix S1).
- Pore size: pore diameter estimated from single‐channel conductance values (Supplementary Methods Table S1).

### 4.13 Liposome preparation for relaxivity measurements

All phospholipids were obtained from Avanti Polar Lipids. Gd‐HPDO3A was kindly provided by Bracco Imaging S.p.A (Colleretto Giacosa [TO], Italy). Large unilamellar vesicles (LUVs) were prepared via the thin lipid film hydration method. Approximately 10 mg of lipids were dissolved in chloroform, and the solvent was slowly evaporated to form a dry film. The film was then hydrated at 55°C with an aqueous solution containing 40 mM Gd‐HPDO3A. The resulting multilamellar vesicle (MLV) suspension was extruded six times through polycarbonate membranes (200 nm pore size) using a Lipex extruder (Northern Lipids Inc.), yielding LUVs. Free (non‐encapsulated) metal complex was removed by exhaustive dialysis against iso‐osmolar (40 mOsm) HEPES/NaCl buffer at 4°C. The size and the polydispersity of the resulting vesicles were assessed by dynamic light scattering using a Zetasizer NanoZS (Malvern Panalytical). The polydispersity index (PDI) of all preparations was below 0.1, confirming homogeneous vesicle populations.

### 4.14 Relaxivity measurements

Proton longitudinal relaxation times (*T*_1_) of the liposome suspensions were measured at 0.47 T and 25°C using a Spinmaster spectrometer (Stelar). Measurements were performed using a standard inversion recovery pulse sequence (180°‐*τ*‐90°). Temperature control was achieved using a VTC‐91 air heater, with the temperature verified inside the probehead using a copper‐constantan thermocouple, yielding a measurement uncertainty of ±0.1°C.

### 4.15 Cell viability assay on HeLa cultured cells

Cell viability was assessed using a LIVE/DEAD fluorescence assay based on calcein‐AM and ethidium homodimer‐1. Calcein‐AM is converted by intracellular esterases into a green‐fluorescent product in live cells, whereas ethidium homodimer‐1 penetrates cells with compromised membranes and binds to nucleic acids, emitting red fluorescence.

HeLa cells were grown to confluence in 96‐well plates in Dulbecco's Modified Eagle Medium (DMEM). In duplicate wells, cells were treated with 20 μM solutions of WT Ub, E16V, E18V, or synthetic heptapeptides Ub1 (TITLEVE), Ub2 (TITLVVE), and Ub3 (TITLEVV). A water‐only control was included. After 24 h incubation at 37°C in a humidified 5% CO_2_ atmosphere, cells were stained with calcein‐AM and ethidium homodimer‐1 for 30 min at room temperature.

Fluorescence images were acquired using an inverted Nikon fluorescence microscope equipped with a 40× objective. Quantification was performed using ImageJ (Schindelin et al., 2012) by separately analyzing the red and green fluorescence channels from two independent fields of view for each condition. Cell death was expressed as the percentage of ethidium‐positive cells relative to the total cell population, calculated as red/(red + green) × 100. Data are reported as mean ± SD.

## AUTHOR CONTRIBUTIONS

Conceptualization: F.A., D.M., R.M.; Methodology: F.A., D.M., R.M.; Investigation: D.M., R.M., V.C., M.L., V.M.; Formal analysis: D.M., R.M., V.C., M.L., V.M.; Writing – original draft: V.C., V.M.; Writing – review & editing: F.A., D.M., R.M.; Supervision and Funding acquisition: F.A. All authors have read and agreed to the published version of the manuscript.

## CONFLICT OF INTEREST STATEMENT

The authors declare no conflicts of interest.

## Supporting information


**TABLE S1:** Characteristic parameters of E16V channels in palmitoyl‐oleoyl‐phosphatidylserine (POPS) planar lipid membranes.
**TABLE S2:** Characteristic parameters of E16V channels at different protein concentrations.
**FIGURE S1:** β‐aggregation propensity and cellular effects of the E16V mutation.
**FIGURE S2:** Structural stability of wild‐type ubiquitin and the E18V variant in the presence of anionic liposomes.
**FIGURE S3:** Liposome‐dependent oligomerization of E16V compared with wild‐type ubiquitin.
**FIGURE S4:** Reversibility of E16V lipid‐induced β‐aggregation by detergent treatment.
**FIGURE S5:** E16V‐induced enhancement of liposome water permeability.
**FIGURE S6:** Voltage‐dependent step‐channel activity of E16V in planar lipid membranes.
**FIGURE S7:** Current–voltage (*I*–*V*) relationship of the E16V channel in POPS planar lipid membranes.
**FIGURE S8:** Concentration‐dependent channel activity of E16V in POPS planar lipid membranes.
**Data S1:** Supplementary Methods.

## Data Availability

The data that supports the findings of this study are available in the supplementary material of this article.
