# Supplementary material for: An edge β‐strand mutation turns ubiquitin into a pore‐forming amyloid
Source: Protein Sci. 2026 May 17;35(6):e70603. doi: 10.1002/pro.70603 (PMC13181154; doi:10.1002/pro.70603)
Supplement: Supplementary file 1 — TABLE S1: Characteristic parameters of E16V channels in palmitoyl‐oleoyl‐phosphatidylserine (POPS) planar lipid membranes. TABLE S2: Characteristic parameters of E16V channels at different protein concentrations. FIGURE S1: β‐aggregation propensity and cellular effects of the E16V mutation. FIGURE S2: Structural stability of wild‐type ubiquitin and the E18V variant in the presence of anionic liposomes. FIGURE S3: Liposome‐dependent oligomerization of E16V compared with wild‐type ubiquitin. FIGURE S4: Reversibility of E16V lipid‐induced β‐aggregation by detergent treatment. FIGURE S5: E16V‐induced enhancement of liposome water permeability. FIGURE S6: Voltage‐dependent step‐channel activity of E16V in planar lipid membranes. FIGURE S7: Current–voltage (I–V) relationship of the E16V channel in POPS planar lipid membranes. FIGURE S8: Concentration‐dependent channel activity of E16V in POPS planar lipid membranes. Data S1: Supplementary Methods. [file PRO-35-e70603-s001.pdf]

SUPPLEMENTARY MATERIAL

An edge β-strand mutation turns ubiquitin into a pore-forming amyloid

Daniela Meleleo[1 †], Rosanna Mallamaci[2 †], Vincenza Calò[3], Maurizio Losacco[3], Vincenzo Mangini[4], Fabio Arnesano[3 *]

[1] Department of Science of Agriculture, Food, Natural Resources and Engineering, University of Foggia, 71122 Foggia, Italy

[2] Department of Biosciences, Biotechnologies and Environment, University of Bari "Aldo Moro", 70125 Bari, Italy

[3] Department of Chemistry, University of Bari "Aldo Moro", 70126 Bari, Italy

[4] Institute of Crystallography, National Research Council, 70126 Bari, Italy

† These authors contributed equally to this work.

* *Corresponding author*: Fabio Arnesano, Via Edoardo Orabona 4, 70126 Bari (Italy). Phone: +39 080 5442768; Fax: +39 080 5442230; e-mail: fabio.arnesano@uniba.it

Supplementary Methods

Pore size estimation

Pore size was estimated from the mean conductance values (Λ_c) obtained by Gaussian fitting of amplitude histograms derived from single-channel current recordings.

Under the standard cylindrical pore approximation, the channel conductance is given by:

$$\Lambda_c = \frac{\sigma \pi r^2}{d} \qquad \text{(Equation S1)}$$

where σ is the specific conductivity of the filling solution (KCl 1 M, σ = 11.2 S m^{-1}) and d is the channel length, assumed to be 5 nm (corresponding to membrane thickness).

Rearranging Equation S1 yields the pore radius:

$$r = \sqrt{\frac{\Lambda_c d}{\sigma \pi}}$$

This expression was used to estimate pore size for each conductance population.

C*hannel lifetime analysis*

Open-state residence time distributions were analyzed to extract kinetic parameters.

Cumulative open-time distributions were constructed by counting the number of channel openings with lifetimes equal to or longer than a given time t (i.e., within the interval t to $t + \Delta t$).

The decay of the cumulative distributions was fitted using a one- or two-exponential function:

$$N(t) = A_1 e^{-t/\tau_1} + A_2 e^{-t/\tau_2} \qquad \text{(Equation S2)}$$

where $N(t)$ is the number of channels remaining open for a time $\geq t$, A_1 and A_2 are the zero-time amplitudes, and τ_1 and τ_2 correspond to the fast and slow kinetic components, respectively.

A single-exponential behavior is obtained when $A_2 = 0$.

An F-test (GraphPad Prism™, version 3.0) was used to determine whether single- or double-exponential models provided the best fit to the experimental data.

Current-voltage relationship

To determine the current-voltage (I-V) relationship, E16V (1 μM final concentration) was added to the *cis* compartment. After detection of a stable channel event at an applied voltage of +80 mV, the transmembrane potential was decreased stepwise in 20 mV increments down to −100 mV.

Channel current amplitudes were plotted as a function of the applied transmembrane potential and analyzed by linear regression.

Kinetic analysis of E16V channels

Open probability and conductance populations

Table S1 reports the central conductance values (Λc) and open probabilities (P_o) for the two conductance populations of E16V channels at all applied voltages.

The open probability P_o represents the fraction of time the channel remains in the conductive state and was calculated by dividing the cumulative open time by the total observation time.

For the first conductance population (Level 1), P_o values are generally higher at positive voltages (except at +80 mV), indicating a longer residence time in the open state under positive bias. Conversely, P_o values for the second population (Level 2), are higher at negative voltages.

The voltage-dependent distribution of P_o suggests asymmetry in the stability of the two conductive states.

Channel lifetimes

Because P_o alone does not provide information on channel kinetics, open-state residence time distributions were analyzed to extract kinetic parameters.

Open-time distributions were fitted using Equation S2, yielding two characteristic time constants (τ_1 and τ_2), corresponding to fast and slow kinetic components, respectively. The resulting values are reported in Table S1.

At positive voltages, both kinetic components are present, whereas at negative voltages the fast component dominates. This indicates greater kinetic stability of E16V channels under positive voltage conditions.

Channel turnover

Channel turnover in planar lipid membranes was assessed through the opening frequency (F), defined as the average number of channel events occurring within a 60 s interval.

As reported in Table S1, F remains approximately constant across the tested voltages, indicating that E16V channel turnover is largely voltage-independent.

Table S1. Characteristic parameters of E16V channels in palmitoyl-oleoyl-phosphatidylserine (POPS) planar lipid membranes. The table reports the mean conductance values (Λ_c±SE) obtained from Gaussian fitting, the open probability (P_o), the channel opening frequency (F±SD), and the channel lifetimes (τ) at different applied voltages. Levels 1 and 2 correspond to the two distinct channel populations described in the text. The number of analyzed channel events (N) ranged from 150 to 330 per condition, out of a total number of 1361 events (Nt).

	Level 1		Level 2				
Vs (mV)	Λ_c±SE (nS)	P_o	Λ_c±SE (nS)	P_o	F±SD	τ_1 (s)	τ_2 (s)
+80	0.035±0.001	0.29	0.299±0.001	0.21	4.16±0.23	1.90	11.92
+60	0.050±0.001	0.51	0.269±0.002	0.20	5.25±0.43	2.21	37.60
+40	0.052±0.0008	0.60	0.279±0.004	0.19	5.76±0.36	1.39	15.29
−40	0.037±0.003	0.10	0.107±0.005	0.62	10.41±0.74	3.63	
−60	0.025±0.003	0.15	0.119±0.003	0.58	5.52±0.44	2.36	
−80	0.024±0.001	0.46	0.114±0.0009	0.36	7.94±0.46	4.51	

Table S2. Characteristic parameters of E16V channels at different protein concentrations. The mean conductance fitted by Gaussian distribution (Λ_c±SE), the open probability (P_o), the frequency (F±SD), the lifetime (τ) of the E16V channels at applied voltage of 80 mV in POPS planar lipid membranes. Levels 1, 2 and 3 represent the three populations of channels. The number of analyzed channel events (N) ranged from 294 to 1387 per condition, out of a total number of 2367 events (Nt).

	Level 1		Level 2		Level 3				
[E16V] (μM)	Λ_c±SE (nS)	P_o	Λ_c±SE (nS)	P_o	Λ_c±SE (nS)	P_o	F±SD	τ_1 (s)	τ_2 (s)
0.01	0.032±0.002	0.04					1.69±0.09	1.07	
0.1	0.029±0.001	0.27					2.64±0.11	1.03	9.83
1	0.035±0.001	0.29	0.299±0.001	0.21			4.16±0.23	1.90	11.92
3	0.030±0.003	0.06	0.307±0.002	0.13	0.484±0.002	0.06	6.91±0.18	0.54	4.23

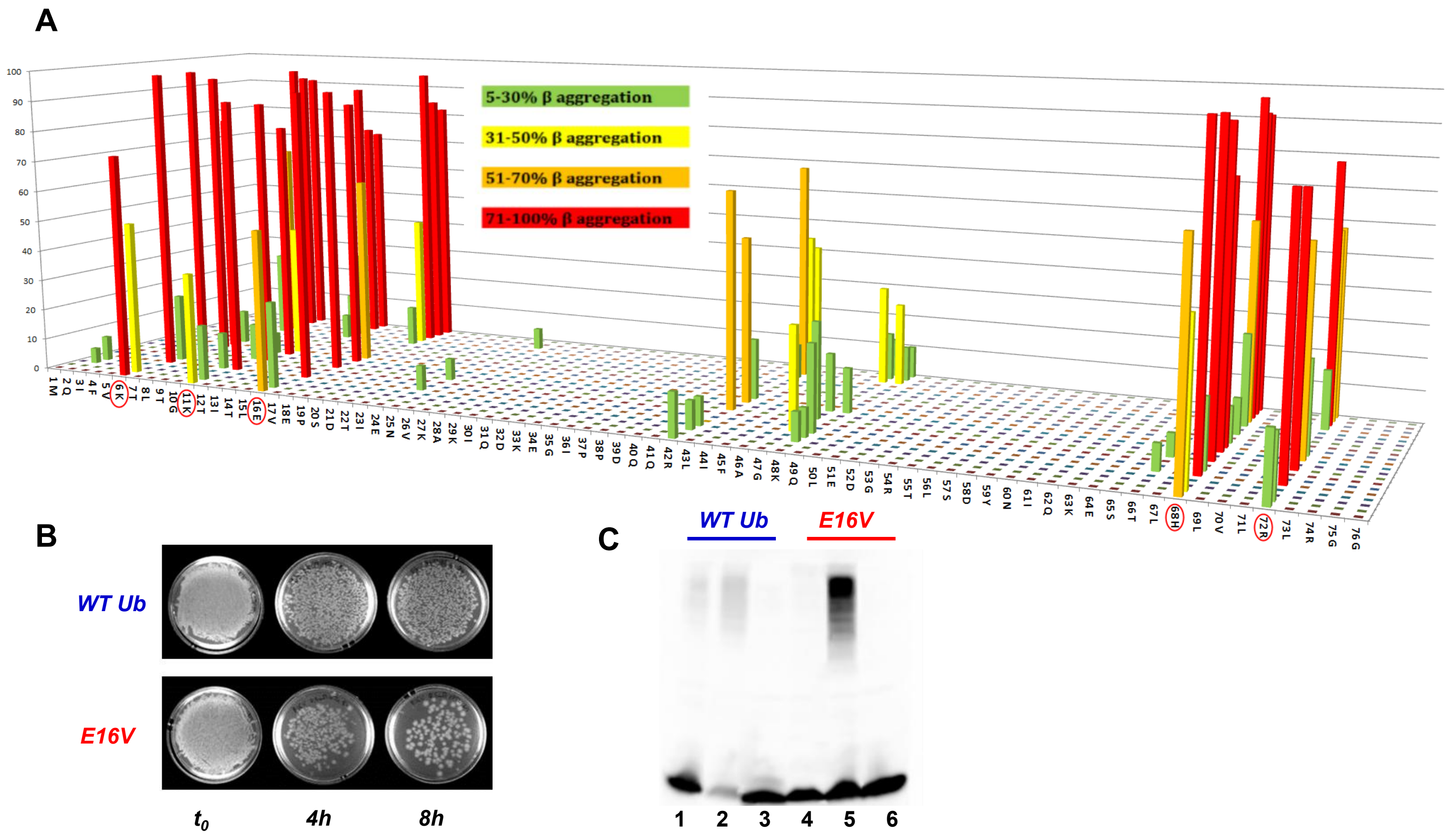

Figure S1. β-aggregation propensity and cellular effects of the E16V mutation. (A) Predicted β-aggregation propensity for all single-point mutants of human ubiquitin (Ub), calculated using the TANGO algorithm. The x-axis represents the Ub amino-acid sequence (76 residues), the y-axis lists all possible amino-acid substitutions, and the color scale indicates the predicted aggregation propensity. (B) Cell viability of *E. coli* expressing wild-type (WT) Ub or the E16V mutant following induction with 1 mM IPTG, monitored at 0, 4, and 8 h post-induction. (C) Western blot analysis of soluble and insoluble fractions from *E. coli* expressing WT Ub or E16V. Lane assignments: lane 1, WT Ub without IPTG; lanes 2–3, insoluble and soluble fractions of WT Ub after induction; lane 4, E16V without IPTG; lanes 5–6, insoluble and soluble fractions of E16V after induction.

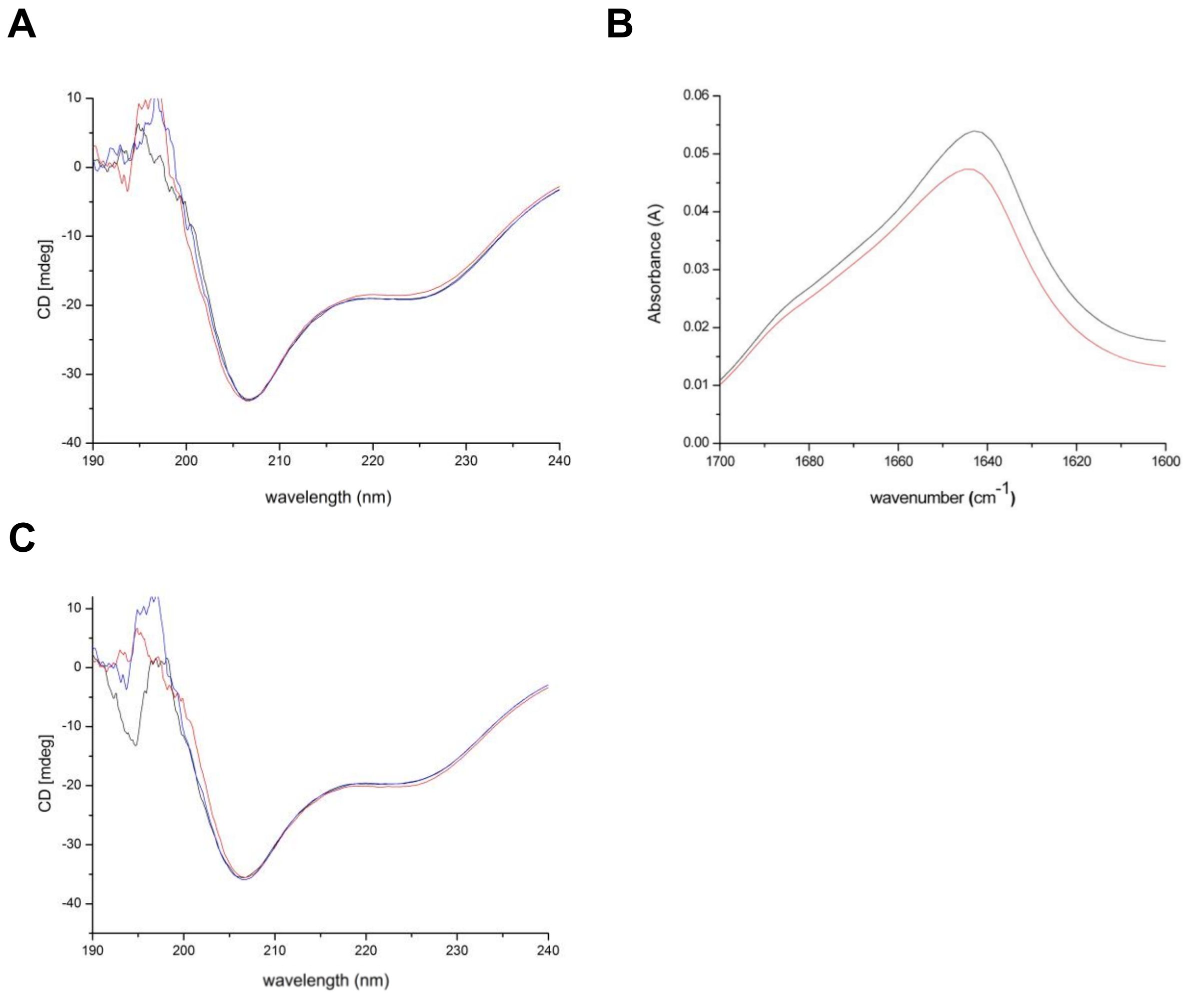

Figure S2. Structural stability of wild-type ubiquitin and the E18V variant in the presence of anionic liposomes. (A) Far-UV circular dichroism (CD) spectra of WT Ub (10 μM) incubated with phosphatidylserine (PS) liposomes (800 μM) in phosphate buffer (pH 7.4) at 37 °C, recorded at time 0 (black), 24 h (blue), and 96 h (red). (B) ATR-FTIR spectra in the amide I region of WT Ub alone (black) and after 96 h incubation with PS liposomes (red) under the same conditions. (C) Far-UV CD spectra of the E18V variant incubated with PS liposomes under identical conditions, recorded at time 0 (black), 24 h (blue), and 96 h (red). In contrast to E16V, no significant time-dependent spectral changes are observed, indicating the absence of liposome-induced conformational conversion.

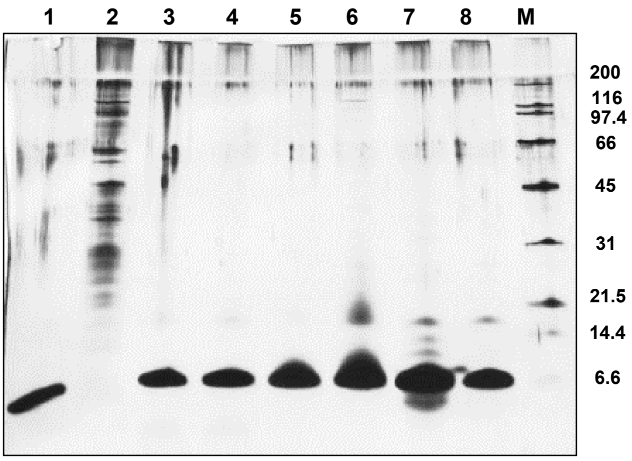

Figure S3. Liposome-dependent oligomerization of E16V compared with wild-type ubiquitin. SDS–PAGE analysis of E16V and WT Ub (10 μM) incubated with phospholipid liposomes (800 μM). Lanes 1–2: E16V + PS liposomes at time 0 and 96 h; lanes 3–4: E16V + phosphatidylcholine (PC) liposomes at time 0 and 96 h; lanes 5–6: WT Ub + PS liposomes at time 0 and 96 h; lanes 7–8: WT Ub alone at time 0 and 96 h; M, molecular weight markers. Time-dependent accumulation of higher-molecular-weight species is observed only for E16V in the presence of PS liposomes.

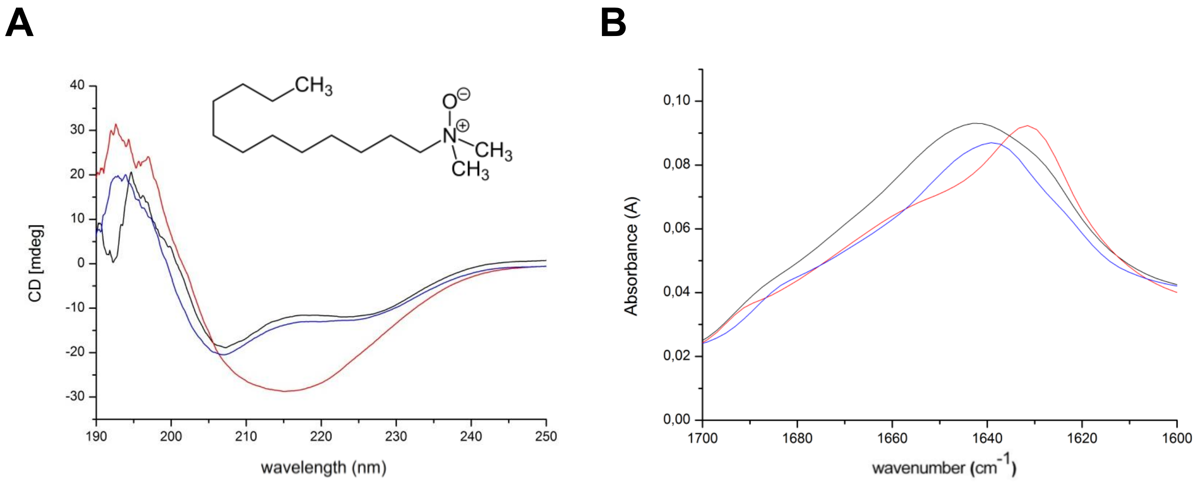

Figure S4. Reversibility of E16V lipid-induced β-aggregation by detergent treatment. (A) Far-UV circular dichroism (CD) spectra of E16V (10 µM) incubated with PS liposomes (800 µM) in phosphate buffer (pH 7.4) at 37 °C at time 0 (black), after 96 h (red), and following addition of 2 mM lauryldimethylamine oxide (LDAO; blue). The chemical structure of LDAO is shown in the inset. (B) ATR-FTIR spectra in the amide I region of E16V alone (black), after 96 h incubation with PS liposomes (red), and after addition of LDAO (blue).

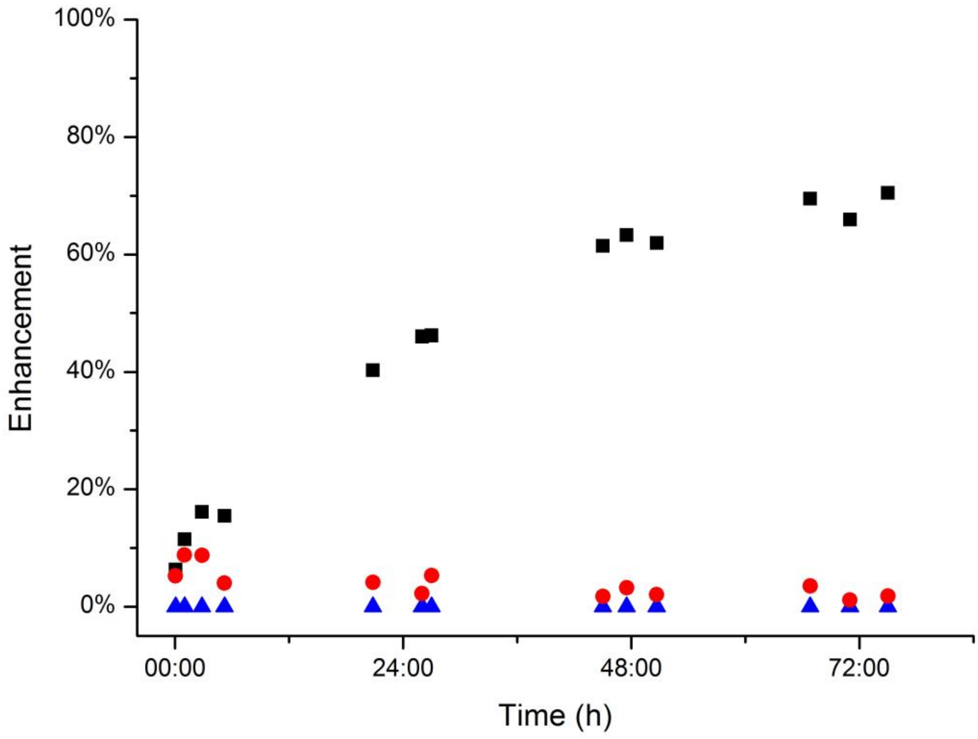

Figure S5. E16V-induced enhancement of liposome water permeability. Time-dependent increase in water permeability of dipalmitoylphosphatidylcholine (DPPC)/dipalmitoylphosphatidylserine (DPPS) (1:1) liposomes incubated with E16V (black squares), WT Ub (red circles), or without protein (blue triangles). Permeability was monitored as the relative increase in the longitudinal water relaxation rate (R_{1p}) measured using encapsulated Gd-based paramagnetic probes. Data are expressed as percentage change relative to liposomes alone at time zero. Only E16V induces a progressive increase in water permeability under the conditions tested. Experimental conditions: [phospholipids] = 20 mg/mL, [protein]:[phospholipids] = 1:80, [EDTA] = 10 mM, T = 25°C, magnetic field = 0.47 T.

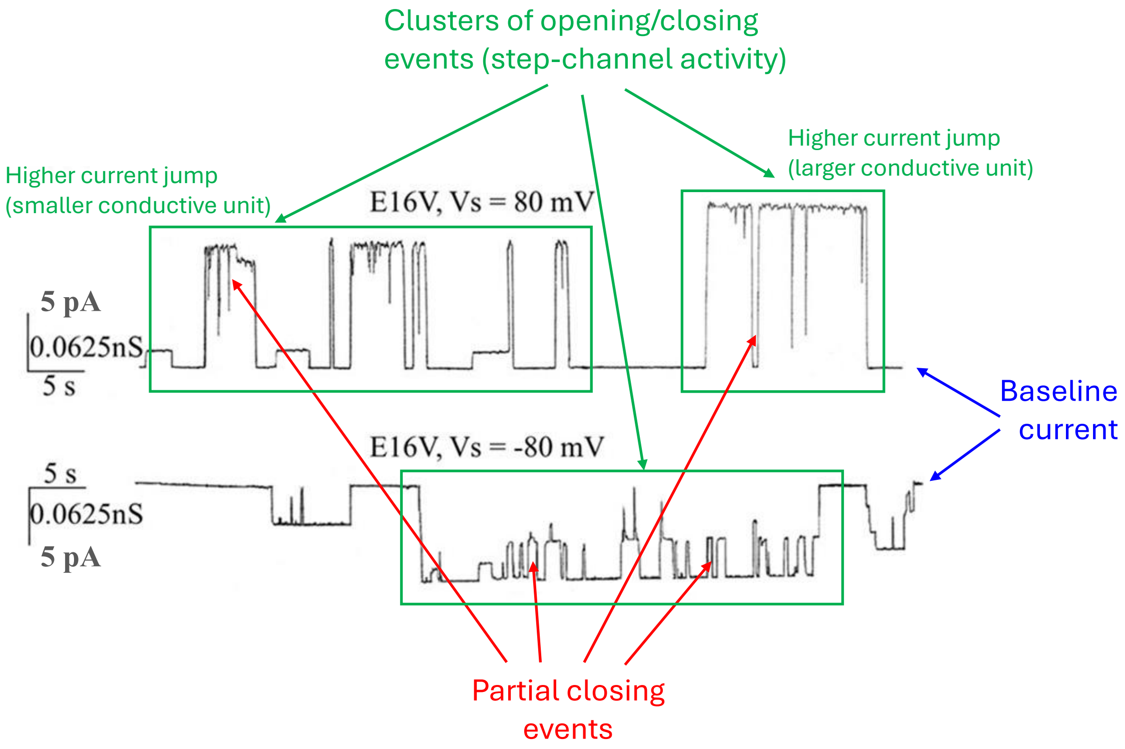

Figure S6. Voltage-dependent step-channel activity of E16V in planar lipid membranes. Representative single-channel current recordings of E16V in POPS planar lipid membranes recorded at +80 mV (*top*) and −80 mV (*bottom*). Discrete opening and closing events characteristic of step-channel activity are observed. At +80 mV, clusters of events with distinct current amplitudes are evident, whereas at −80 mV partial closures and less regular current levels are observed, indicating asymmetric channel behavior. Baseline current and conductance levels are indicated.

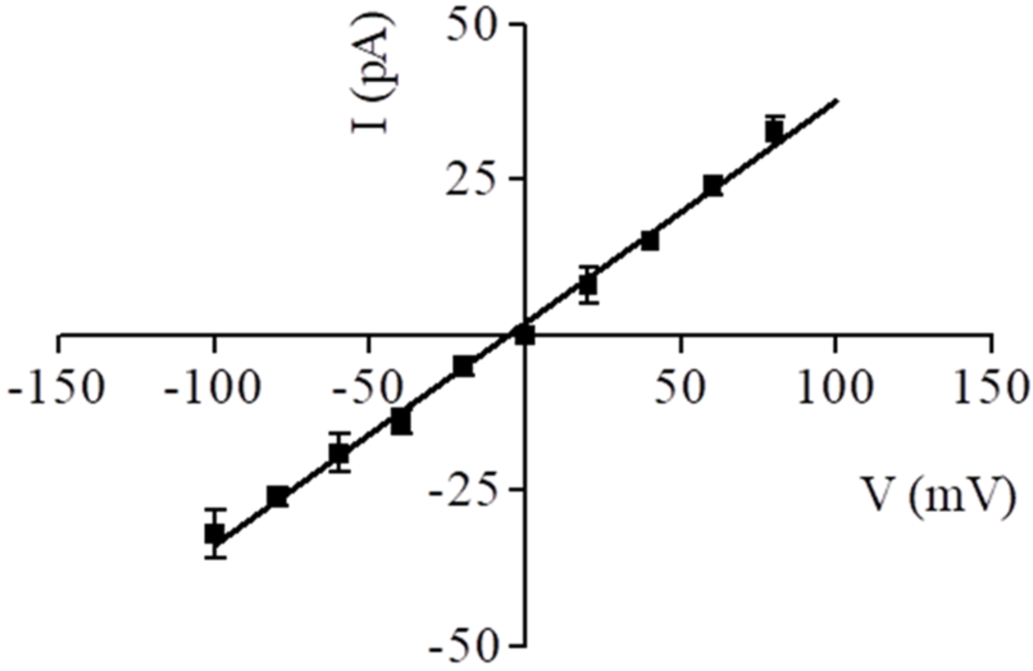

Figure S7. Current–voltage (I–V) relationship of the E16V channel in POPS planar lipid membranes. The linear dependence of current on applied voltage indicates ohmic behavior. Each data point represents the mean ± SD of at least five single-channel current measurements. Experimental conditions: 1 M KCl (pH 7.0), 23 ± 1 °C.

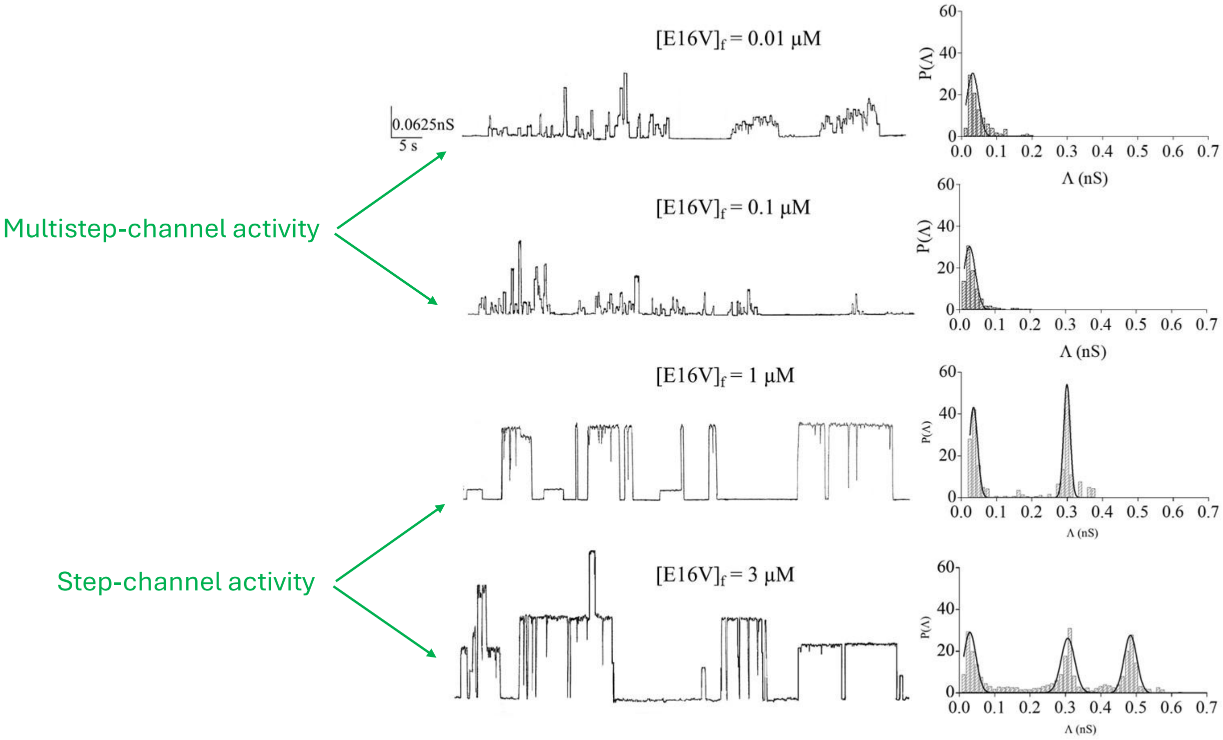

Figure S8. Concentration-dependent channel activity of E16V in POPS planar lipid membranes. Representative single-channel current traces (*left*) and corresponding conductance histograms (*right*) recorded at increasing E16V concentrations ($[E16V]_f$ = 0.01, 0.1, 1, and 3 µM). The probability histograms, P(Λ) were fitted with a mixture of Gaussian functions (solid curves) to resolve distinct conductance populations. At low concentrations (0.01 and 0.1 µM), *multistep-channel activity* is observed, characterized by multi-level current fluctuations reflecting adsorption, insertion, and progressive assembly of conductive units within the membrane. At higher concentrations (1 and 3 µM), the behavior shifts to *step-channel activity*, displaying abrupt and discrete current transitions consistent with insertion of preassembled oligomeric units into the membrane and formation of more stable conductive structures. Experimental conditions: 1 M KCl (pH 7.0), applied voltage Vs = +80 mV, T = 23 ± 1 °C.
